# Identification, characterization and antigenicity of the *Plasmodium vivax *rhoptry neck protein 1 (*Pv*RON1)

**DOI:** 10.1186/1475-2875-10-314

**Published:** 2011-10-24

**Authors:** Darwin A Moreno-Perez, Marjorie Montenegro, Manuel E Patarroyo, Manuel A Patarroyo

**Affiliations:** 1 Fundación Instituto de Inmunología de Colombia (FIDIC), Carrera 50 No. 26-20, Bogotá, Colombia; 2 Universidad del Rosario, Calle 63D No. 24-31, Bogotá, Colombia; 3 Universidad Nacional de Colombia, Carrera 45 No. 26-85, Bogotá, Colombia

**Keywords:** Rhoptry, *Plasmodium vivax*, Antigenicity, vaccine candidate

## Abstract

**Background:**

*Plasmodium vivax *malaria remains a major health problem in tropical and sub-tropical regions worldwide. Several rhoptry proteins which are important for interaction with and/or invasion of red blood cells, such as *Pf*RONs, *Pf*92, *Pf*38, *Pf*12 and *Pf*34, have been described during the last few years and are being considered as potential anti-malarial vaccine candidates. This study describes the identification and characterization of the *P. vivax *rhoptry neck protein 1 (*Pv*RON1) and examine its antigenicity in natural *P. vivax *infections.

**Methods:**

The *Pv*RON1 encoding gene, which is homologous to that encoding the *P. falciparum *apical sushi protein (ASP) according to the plasmoDB database, was selected as our study target. The *pvron1 *gene transcription was evaluated by RT-PCR using RNA obtained from the *P. vivax *VCG-1 strain. Two peptides derived from the deduced *P. vivax *Sal-I *Pv*RON1 sequence were synthesized and inoculated in rabbits for obtaining anti-*Pv*RON1 antibodies which were used to confirm the protein expression in VCG-1 strain schizonts along with its association with detergent-resistant microdomains (DRMs) by Western blot, and its localization by immunofluorescence assays. The antigenicity of the *Pv*RON1 protein was assessed using human sera from individuals previously exposed to *P. vivax *malaria by ELISA.

**Results:**

In the *P. vivax *VCG-1 strain, RON1 is a 764 amino acid-long protein. *In silico *analysis has revealed that *Pv*RON1 shares essential characteristics with different antigens involved in invasion, such as the presence of a secretory signal, a GPI-anchor sequence and a putative sushi domain. The *Pv*RON1 protein is expressed in parasite's schizont stage, localized in rhoptry necks and it is associated with DRMs. Recombinant protein recognition by human sera indicates that this antigen can trigger an immune response during a natural infection with *P. vivax*.

**Conclusions:**

This study shows the identification and characterization of the *P. vivax *rhoptry neck protein 1 in the VCG-1 strain. Taking into account that *Pv*RON1 shares several important characteristics with other *Plasmodium *antigens that play a functional role during RBC invasion and, as shown here, it is antigenic, it could be considered as a good vaccine candidate. Further studies aimed at assessing its immunogenicity and protection-inducing ability in the *Aotus *monkey model are thus recommended.

## Background

Malaria remains one of the prevailing health problems worldwide. According to the World Health Organization (WHO) [1], nearly 225 million people are infected annually; about 785,000 of them die as a direct consequence of this disease, of which 85% are children aged less than five years. Although malaria in humans is caused by *Plasmodium falciparum, Plasmodium vivax, Plasmodium ovale, Plasmodium malariae *and *Plasmodium knowlesi*, the first two species represent about 90% of the clinical cases reported [2]. *P. falciparum *is responsible for causing the disease's highest mortality rates while *P. vivax *represents significant morbidity having socioeconomic implications [3]. In spite of international control strategies and policies having been implemented during the last fifty years, mortality figures are still alarming; therefore, developing an efficient vaccine to combat this imminent threat has become an urgent need.

Invasion of red blood cells (RBC) by *Plasmodium *parasites involves highly coordinated events which are directed by a set of proteins secreted from the apical organelles (rhoptries and micronemes) [4]. It has been shown that several rhoptry proteins, such as *Pf*RON2, -4, and the *Pf*AMA-1 antigen (secreted by micronemes), are involved in tight junction formation between the parasite and its target cell [5-7]; it has also been found that some others (such as *Pf*RON1, *Pf*92, *Pf*38, *Pf*12 and *Pf*34) are associated with detergent-resistant membrane microdomains (DRM) through glycosylphosphatidylinositol (GPI)-anchor sequences [8], which are considered organizing centers for the assembly of molecules implicated in cell signaling [9,10]. To date, several of these DRM proteins have been shown to play an active role in host cell interaction and to trigger antibody responses in the host [11-15].

Rhoptry neck protein 1 (RON1), initially described in *Toxoplasma gondii *(*Tg*RON1) [16], has been a particularly interesting protein. It is a highly-conserved antigen amongst *Apicomplexa *members. Different *tgron1 *homologous genes have also been found in members of the *Plasmodium *genus, such as *P. falciparum *[16,17]. *Pf*RON1 is also known as the apical sushi protein (ASP), exhibiting a prominent transcriptional peak towards the end of the intraerythrocyte lifecycle [17]. This protein has 731 amino acids encoded by 4 exons and ~85.46 kDa molecular mass. It has been previously demonstrated that *Pf*ASP undergoes proteolytic processing, resulting in ~50 kDa and ~30 kDa polypeptides [18]. The protein also contains a signal peptide, a GPI-anchor sequence and a complement control protein (CCP) or sushi-like conserved domain [19]; this domain typically contains ~60 residues and represents a protein module involved in protein-protein interactions and/or cell adhesion, containing 4 cysteines, proline residues and highly conserved acidic amino acids (aspartic and glutamic acid). Furthermore, it has been recently found that *Pf*RON1 has 4 high activity binding peptide sequences (HABPs) to erythrocytes (manuscript in preparation), and could thus be important for parasite entry to target cells.

In the search for an effective anti-malarial vaccine several studies have been focused on identifying antigens expressed during the parasite's asexual stage due to the fact that some of these antigens are mainly responsible for invasion of RBC [20,21]. Several proteins included in developing a promising vaccine against *P. falciparum *have been functionally characterized to date [21]. However, studies carried out with *P. vivax *have been limited due to the difficulty in maintaining an *in vitro *continuous parasite culture. Taking this into account and considering the degree of conservation observed between the genetic content of different *Plasmodium *species [23,24], some bioinformatics tools have been used for homology searches of the complete *P. vivax *genome for antigens already described in *P. falciparum*. To date, a considerable number of surface [25-28] and rhoptry proteins [22,29-35] have been experimentally identified and characterized in *P. vivax *using both its genome [21] and intraerythrocyte lifecycle transcriptome [36] as well as an *Aotus*-adapted strain [37]. These proteins belong to a list of antigens, which are being tested as vaccine candidates in the *Aotus *monkey model.

This study shows the identification and characterization of the *Pv*RON1 protein which is homologous to *Pf*ASP. Different aspects such as *Pv*RON1 transcription and expression at the end of the intraerythrocyte life cycle, the subcellular localization pattern and the ability to trigger an immune response in patients who have presented active episodes of *P. vivax *malarial infection have been determined by molecular biology and immunochemistry techniques.

## Methods

### Bioinformatics analysis

The *P. vivax ron1 *gene sequence (which is homologous to the *Pf*ASP encoding gene) was obtained from the plasmoDB database [38]; likewise, *pfasp *orthologous genes in other malarial species were searched. Identity and similarity values between *P. falciparum - P. vivax *and *P. vivax - P. knowlesi *hypothetical protein products were determined using ClustalW software [39]. The presence of a signal peptide, repeat sequences and a GPI-anchor sequence was determined by SignalP 3.0 [40], XSTREAM repeat [41] and GPI-anchor [42] bioinformatics tools, respectively. Putative domains were assessed using the Interpro database [43]. Antheprot software [44] was used for determining two peptides throughout the *Pv*RON1 hypothetical sequence (PlasmoDB accession number: PVX_000945) that could be good B-cell epitopes, taking the highest average hydrophilicity (>4.9), solvent accessibility (>162) and Parker's antigenicity (>37.8) values into account (calculated in a 20 residue sliding window), according to previously established parameters [45-47].

### Animal handling

The experimental handling of animals used here was carried out in accordance with Colombian Law 84/1989 and resolution 504/1996. *Aotus *monkeys kept at FIDIC's primate station (Leticia, Amazon) and New Zealand rabbits provided by the Instituto Nacional de Salud (Bogotá, Colombia) were handled following the guidelines for the care and use of laboratory animals (National Institute of Health, USA) under the constant supervision of a veterinarian. Immunization and bleeding procedures for *Aotus *monkeys had been previously approved by our institute's ethics committee, and were carried out in agreement with the conditions stipulated by CorpoAmazonia (resolution 00066, September 13^th^2006). A monkey from the *Aotus *genus was experimentally infected with the VCG-1 strain (Vivax Colombia Guaviare 1) and subjected to daily monitoring to assess the progress of infection throughout the whole study (up to day 18) using acridine orange staining. The monkey was treated with pediatric doses of chloroquine (10 mg/kg on the first day and 7.5 mg/kg/day until the fifth day) and primaquine (0.25 mg/kg/day from the third to the fifth day) at the end of the study to guarantee total blood parasite clearance. Once experiments were over, CorpoAmazonia officers supervised the primate's return to its natural habitat in excellent health conditions.

### Isolating the *P. vivax *parasite

The VCG-1 strain was obtained according to a previously described methodology [37]. Mature forms of the parasite, mainly schizonts, were purified from a blood sample (3 mL) infected with *P. vivax *using a Percoll discontinuous gradient (GE Healthcare, Uppsala, Sweden), according to a previously established protocol [48]. This schizont-enriched sample was then used for performing all of the following procedures: RNA, genomic DNA or parasite total protein extraction, as well as indirect immunofluorescence assays.

### RNA extraction and cDNA synthesis

Total RNA from the schizont-enriched sample was extracted using the Trizol method and treated with RNase-free RQ1 DNase (Promega, Wisconsin, USA), according to the manufacturer's instructions. 5 μL RNA were used to synthesize cDNA by RT-PCR, using the SuperScript III enzyme (Invitrogen, California, USA). Complementary DNA synthesis was carried out in the following conditions: 65°C for 5 mins, 50°C for 1 hour and 70°C for 15 mins. After a 15-min incubation period with RNase (Promega, USA) at 37°C, the product was stored at -20°C until its use.

### Cloning and sequencing

Three specific primer sets were designed to cover the whole gene sequence and produce two smaller-sized recombinant fragments (Forward 5'-ACAGAAGAGAAGAGAACAGA-3', Reverse 5'-GTTCACACATGCGGTCAC-3'; Forward 5'- ATGGCGAAGGAGCCCAAGTG -3', Reverse 5'- ATCCCTAGCAATGCTTCG -3'; Forward 5'-ATGCTGCTAGTGCCACCCG-3', Reverse 5'- CTGAACACCATCGAAATCG -3'). After PCR amplification using the Platinum *Pfx *DNA polymerase enzyme (Invitrogen, California, USA), each product was purified by Wizard PCR preps kit (Promega), ligated to the pEXP5 CT/TOPO vector (Invitrogen) and cloned in *Escherichia coli *TOP10 bacteria (Invitrogen), following the manufacturer's instructions. Recombinant DNA was purified using an UltraClean mini plasmid prep purification kit (MO BIO laboratories, California, USA) and each sequence's integrity was confirmed by sequencing two clones obtained from independent PCRs, using the automated genetic analyzer ABI PRISM 310 (PE Applied Biosystems, California, USA).

### Peptide synthesis and polyclonal antibody production

Two 20-amino-acid-long peptides designed on the *Pv*RON1 deduced sequence (Sal-1 strain) (CG-KHKEGGAAKRHKKEPHEQRG-GC and CG-EDASVADKDGQPGERGQDGQ-GC) were synthesized according to a previously established methodology [49], polymerized, lyophilized and characterized by RP-HPLC and MALDI-TOF MS. Afterwards, a 150 μg dose of a mixture from both synthetic peptides, emulsified in Freund's complete adjuvant (FCA) (Sigma, Missouri, USA), was inoculated into New Zealand rabbits on day 0, while the same mixture emulsified in Freund's incomplete adjuvant (FIA) was inoculated on days 21 and 42. Sera were collected before the first immunization (pre-immune sera) and 20 days after the last booster dose (post III sera). Rabbit sera were absorbed with *E. coli *proteins coupled to a Sepharose column and then stored at -20°C until use, according to the manufacturer's instructions (Amersham Biosciences, Buckinghamshire, UK).

### Parasite protein extraction

A parasite pellet was treated with 0.2% saponin, then washed six times with PBS and homogenized in lysis buffer (5% SDS, 10 mM PMSF, 10 mM iodoacetamide, 1 mM EDTA). The total lysate was quantified by using a micro BCA protein assay kit (Thermo scientific) and resolved in SDS-PAGE in reducing and non-reducing conditions. Immunoblot detection was carried out using sera from rabbits previously immunized with synthetic peptides.

### Isolating detergent-resistant microdomains (DRMs)

Parasite mature forms extracted by the Percoll method were treated with 0.2% saponin in PBS for 5 minutes. After six washes in PBS, the pellet was suspended in a 200 μL TNET solution (1% Triton X-100, 25 mM Tris-HCl, 150 mM NaCl and 1 mM EDTA) which contained protease inhibitors (1 mM PMSF, 1 mM iodoacetamide, 1 mM EDTA and 1 mg/mL leupeptin). The sample was poured into two aliquots which were then treated in the following conditions: a 100 μL aliquot was incubated at 4°C for 30 minutes and subsequently centrifuged at 7,550 rpm for 10 minutes; the remaining sample was kept at 37°C for 30 minutes and then centrifuged in the same conditions described above. The supernatant was recovered in a new Eppendorf tube and the remaining pellet from each assay was suspended in lysis buffer (5% SDS, 10 mM PMSF, 10 mM iodoacetamide, 1 mM EDTA). Each fraction was quantified by Micro BCA protein assay kit (Thermo scientific) and resolved in 12% SDS-PAGE.

### Expression and purification of recombinant protein fragments

Once the sequence from cloned inserts had been confirmed, these were transformed in the *E. coli *BL21-AI strain (Invitrogen), according to the manufacturer's recommendations. In brief, cells were grown overnight at 37°C and then inoculated in Terrific broth medium supplemented with 0.1 mg/mL ampicillin and 0.1% (w/v) D-glucose. Once the cells had reached their stationary phase (0.6 OD_600_), 0.2% L-arabinose (w/v) was added as inductor and these were incubated again in the same conditions described above for 4 hours. The culture was centrifuged at 13,000 rpm for 30 minutes and the pellet was suspended in extraction buffer (6 M Urea, 12 mM imidazole, 10 mM Tris-Cl, 100 mM NaH_2_PO_4 _and 10 mg/mL lysozyme) supplemented with protease inhibitors (1 mM PMSF, 1 mM iodoacetamide, 1 mM EDTA and 1 mg/mL leupeptin) and lysed by sonication. r*Pv*RON1-a and r*Pv*RON1-c recombinant fragment expression was assessed on 12% SDS-PAGE. pEXP5 CT/TOPO vector was used to facilitate purification and recognition by monoclonal anti-histidine antibodies. This vector adds a six-histidine tag towards the C-terminus of each fragment, which is used for purifying the recombinant protein by affinity chromatography using a Ni^+2^-NTA resin (Qiagen, California, USA). Briefly, the extraction buffer described above was used to elute the non-retained proteins and then the same buffer plus 500 mM imidazole was used to elute the recombinant protein. All individually collected fractions were analyzed by SDS-PAGE and Western blot. Pure fractions were dialyzed in PBS pH 7.0. The protein was ultrafiltered and concentrated using an Amicon filtration system (LabX scientific marketplace, Midland Canada) and quantified with a micro BCA protein assay kit (Thermo Fisher scientific, Pittsburgh, PA, USA).

### SDS-PAGE and Western blot

5 μg of each recombinant and 50 μg of both parasite total lysate and the fractions obtained in DRMs were separated on SDS-PAGE gels and transferred to nitrocellulose membranes. These membranes were blocked with 5% skimmed milk in 0.05% PBS-Tween for one hour. After three washes with 0.05% PBS-Tween, each membrane was cut into strips which were individually analyzed as follows: strips with recombinant protein were incubated for 1 hour at room temperature in 5% skimmed milk in 0.05% PBS-Tween, including a 1:100 dilution of rabbit (pre-immune and post-III) or human sera. Strips with parasite total lysate and those containing DRM fractions were incubated for 1 hour at room temperature in 5% skimmed milk in 0.05% PBS-Tween, including a 1:100 dilution of anti*-Pv*RON1 antibodies. After 3 washes, strips were incubated for 1 hour with goat anti-rabbit IgG coupled to phosphatase as secondary antibody in a 1:4,500 dilution at room temperature, or goat anti-human IgG in a 1:5,000 dilution. Western blot for recombinant proteins was carried out as follows: a nitrocellulose strip was used as positive control; this strip was incubated with a peroxidase-coupled monoclonal anti-histidine antibody diluted 1:4,500 in skimmed milk plus 0.05% PBS-Tween. Blots were revealed with a substrate peroxidase VIP kit (Vector Laboratories, Burlingame, Canada) or BCIP/NBT color development substrate kits (Promega), according to the manufacturer's instructions.

### Indirect immunofluorescence assay (IFA)

Blood smears from the *Aotus *monkey infected with *P. vivax *were fixed with 4% (*v/v*) formaldehyde. Slides were washed thrice with PBS and blocked at 37°C for 45 minutes using a 1% (*v/v*) bovine serum albumin (BSA) solution in PBS. After 3 washes, slides were incubated at 37°C for 1 hour with a mixture of anti-*Pv*RON1 rabbit sera in 1:30 dilution and anti-*Pv*RhopH3 mice sera (previously obtained in our institute [30]) in 1:40 dilution, suspended in PBS -1% BSA - 0.1% Triton X-100 solution. After three washes, plates were incubated with FITC-conjugated anti-rabbit IgG antibody (Sigma) and Red-conjugated anti-mouse IgG antibody (Millipore) in previously described conditions. Slides were stained with DAPI (2 μg/mL) for 20 minutes at room temperature and then observed under an Olympus BX51 fluorescence microscope, using 100× oil immersion objective.

Accession number: the nucleotide and amino acid sequences used here have been reported in the GenBank database, under accession number JN188400.

## Results and Discussion

### *Pvron1 *gene identification and localization in a syntenic chromosome region

**Figure 1:**
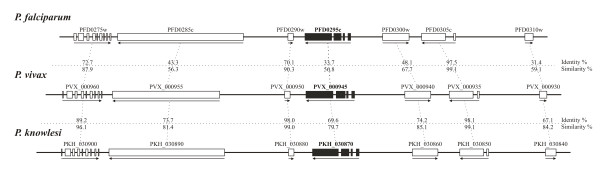
**Schematic scale organization showing the localization of *pfasp *(PFD0295c), *pvron1 *(PVX_000945) and *pkron1 *(PCH_030870) genes within *P. falciparum, P. vivax *and *P. knowlesi *chromosome fragments **. Exon-intron organization, ORF orientation and gene IDs are shown in accordance with the plasmoDB database. Identity (Id) and similarity (S) values between each hypothetical protein product contained in *P. falciparum *- *P. vivax *and *P. vivax *- *P. knowlesi *upstream and downstream contigs are shown.

The initial *P. vivax ron1 *gene sequence selected for this study was found in the plasmoDB database (Accession number PVX_000945). According to the information found there, the *ron1 *gene seemed to be ubiquitous within the *Plasmodium *genus, since orthologous genes were found in the *Plasmodium falciparum, Plasmodium knowlesi *and *Plasmodium berghei *genomes. Additionally, the database showed that open reading frame (ORF) orientation and exon-intron structure were highly conserved among the genes encoding the hypothetical protein products in these parasite species. Such genome organization was consistent with the identity (Id) and similarity (S) values obtained when *P. falciparum *- *P. vivax *and *P. vivax *- *P. knowlesi *contigs were analyzed (Figure 1). It is worth noting that the high degree of S observed between *P. vivax *- *P. knowlesi *was in agreement with the evolutionary proximity found in a previous phylogenetic analysis of conserved regions from the circumsporozoite protein (CSP) encoding gene [50] and in a comparative genomics study of *P. vivax *[23] which showed that these organisms share similar chromosomal regions. Interestingly, the Id value found between the alignment of ORFs encoded by *pfasp *(PFD0295c) and *pvron1 *(PVX_000945) genes was similar to the Id values found in antigens considered good candidates for designing an effective vaccine against *P. vivax *malaria [22,28,29], suggesting that *Pv*RON1 should be an interesting candidate for immunological trials.

### *Pv*RON1 characterization *in silico*

**Figure 2:**
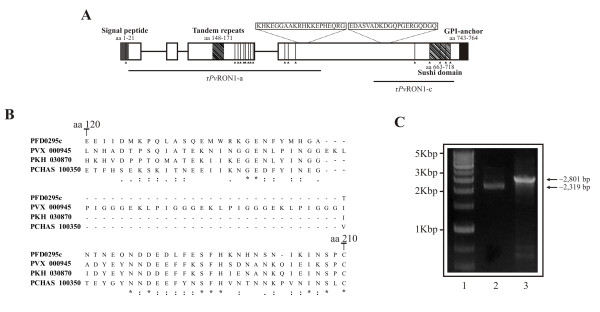
**In silico characterization, alignment of a RON1 protein-fragment in different malarial species and pvron1 gene transcription**. (A) *pvron1 *gene scheme, showing the signal peptide, GPI-anchor sequence, TR and sushi domain localizations. Cysteine residues are represented by lines. Peptides used for rabbit immunization are shown, as well as r*Pv*RON1-a and r*Pv*RON1-c fragments expressed as recombinants. (B) Alignment of a RON1 protein-fragment from *P. falciparum, P. vivax, P. chabaudi *and *P. knowlesi *species. This figure shows the region consisting of amino acids 120 to 210, which includes a repeat sequence which is exclusive for *P. vivax *species. Asterisks indicate identical amino acids. A dot refers to weakly similar amino acids and two dots indicate strongly similar amino acids. (C) *pvron1 *gene amplification by PCR. Line 1 shows the molecular weight marker. Lines 2 and 3 correspond to amplification of genomic and complementary DNA.

The *pvron1 *gene from SAL-1 strain has 2,801 bp; it encodes to a 772 amino acid-long protein (~84.5 kDa estimated molecular weight). This protein is 41 residues longer compared to *Pf*ASP (731 aa) [18]. *Pv*RON1 presents two hydrophobic regions in the N and C-terminus matching a secretory signal sequence composed by the first 21 amino acids and another located between amino acids 743 to 764 (Figure 2A). This latter feature plus the result of the GPI-anchor predictor, point out to the C-terminal anchoring of this protein by GPI. In addition, a sushi-like domain (also known as complement control protein (CCP) module) was predicted; this domain consists of 56 amino acids, four corresponding to cysteines (Cys) which intervene in the domain's structural conformation to mediate protein-protein binding and/or cell adhesion [19].

XSTREAM analysis revealed a tandem repeat (TR) between amino acids 148 to 171 (Figure 2A). According to the alignment performed with other homologous proteins in *Plasmodium *species (*P. falciparum, P. knowlesi *and *P. chabaudi*) this region has been shown to be exclusive for *P. vivax *(Figure 2B). The TR consists of four blocks of eight amino acids having the EKLPIGGG consensus sequence. Antheprot software prediction has shown that this could be a good B-cell lineal epitope. Another two blocks having a similar sequence to that of the TR were found between amino acids 133 to 147; these sequences showed substitutions or deletions of some residues, suggesting that this region might be under selective pressure. It has been previously described that the polymorphic repeat sequences found in some *P. falciparum *malarial antigens, such as the circumsporozoite protein (CSP), the ring-infected erythrocyte surface antigen (RESA) and the S-antigen, can affect antibody affinity maturation and mask critical epitopes involved in invasion from being recognized [51-54]; the present analysis has thus led us to suggesting that the TR regions found in *Pv*RON1 could be part of a mechanism used by the parasite to evade the host's humoral immune response. However, further experimental evidence is needed to confirm this hypothesis.

### The *pvron1 *gene is transcribed during the blood stage

The presence of *ron1 *gene transcripts in the *P. vivax *VCG-1 strain was assessed by PCR, using cDNA as template. Figure 2C shows the amplification products obtained from RT-PCR and genomic DNA, indicating that the *pvron1 *gene is transcribed in schizont-enriched samples. These results agreed with previously reported studies showing a peak transcription from 35 to 40 hours of the *P. vivax *Sal-1 strain intraerythrocyte cycle [36] and between 35 and 48 hours in the *P. falciparum *3D7 strain [17]. Once genomic and complementary DNA sequences were aligned, it was found that the *pvron1 *gene was encoded by four exons, similar to the homologous *pfasp *gene [18], the fourth of them containing a sushi domain. Two nucleotide substitutions and a thirty-two base pair deletion corresponding to a repeat block (EKLPIGGG) were observed when the *pvron1 *gene sequences from Sal-1 reference strain and VCG-1 *Aotus-*adapted strain were compared. These mutations were non-synonymous and produced changes in the following amino acids: glutamic acid for glycine in position 148 and isoleucine for threonine in position 740. Furthermore, 17 Cys residues were shown to be conserved between these two strains. When comparing these residues from the *Pv*RON1 sequence with its *P. falciparum *homologue (*Pf*ASP), these presented an identical localization, suggesting that there was probably a degree of conservation in the 3D structure of such molecules.

### *Pv*RON1 expression and association with DRMs

**Figure 3:**
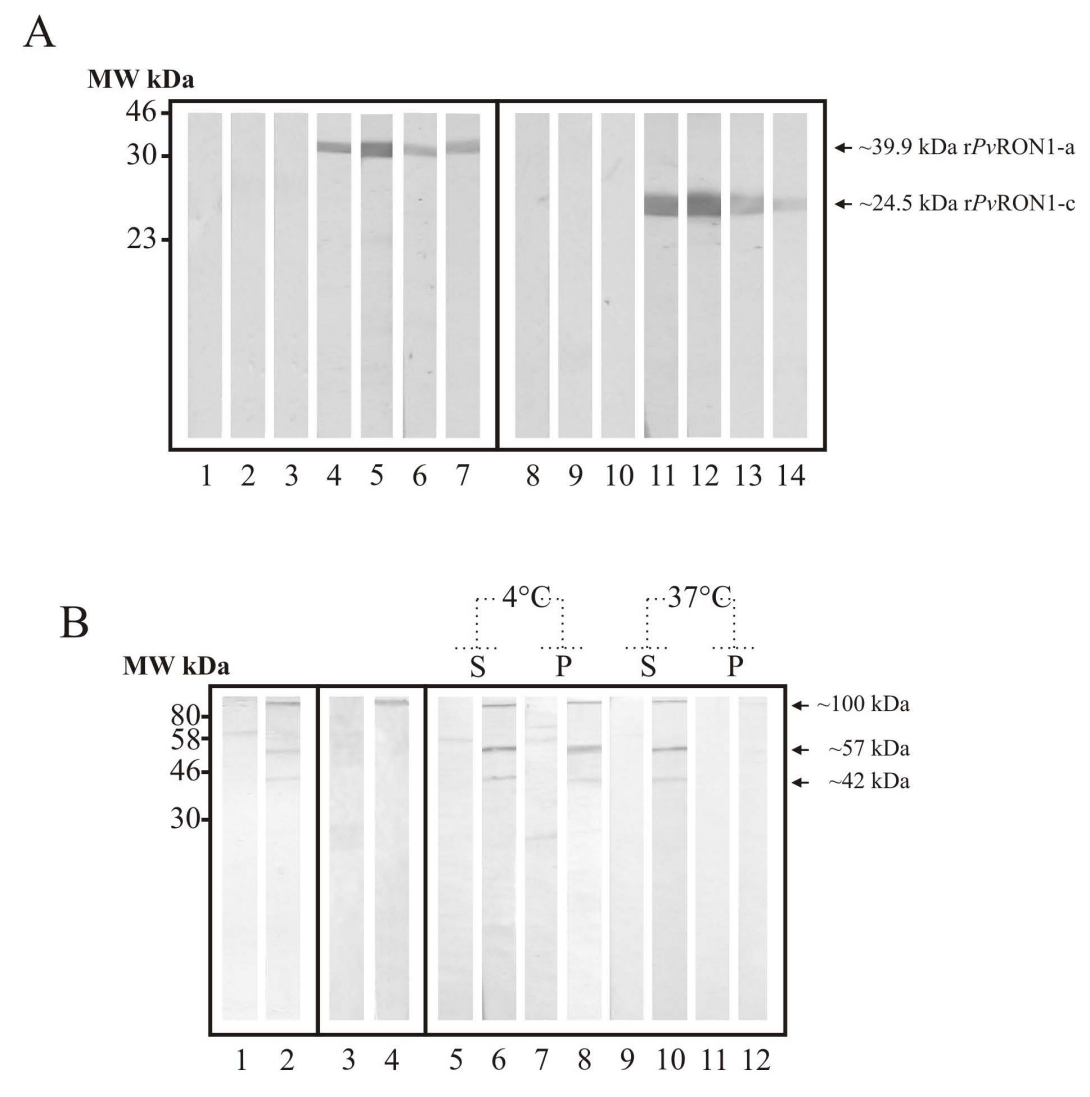
**Detection of PvRON1 recombinant fragments and the native protein by polyclonal antibodies**. (A) Evaluation of anti-*Pv*RON1 polyclonal antibody recognition by Western blot. Lines 1-3 and 8-10 correspond to recognition by pre-immune sera. Lines 4-6 and 11-13 correspond to detection with rabbits' post III sera . Lines 7 and 14 indicate the recognition of purified r*Pv*RON1-a and r*Pv*RON1-c fragments by anti-polyhistidine monoclonal antibody. (B) Detection of the protein in parasite total lysate and DRMs. Lines 1 and 2, parasite lysate in reducing conditions. Lines 3 and 4, parasite lysate in non-reducing conditions. Lines 5-12, DRM fractions treated at 4°C and 37°C. S and P letters represent the supernatant and pellet.

Two peptides derived from the deduced *P. vivax *Sal-I *Pv*RON1 sequence were synthesized and inoculated in rabbits for obtaining anti-*Pv*RON1 antibodies that could recognize the protein in parasite lysate. The antibodies' ability to recognize *Pv*RON1 was assessed by Western blot, using purified r*Pv*RON1-a and r*Pv*RON1-c fragments as antigens (Figure 3A). Immunodetection assays carried out here with polyclonal sera detected bands at ~100 kDa, corresponding to the protein without the signal peptide, and others at ~57 kDa and ~42 kDa, suggesting proteolytic products (Figure 3B; Line 2). Given that the expected weight of the complete protein was slightly lower (~81.35 kDa), it is more likely that the result corresponded to anomalous migration, similar to what occurs with other *Apicomplexa *antigens already identified in the rhoptries, such as *Tg*RON2 [7], *Tg*ROP1 [55] and *Pv*RON2 [30]. When the experiment was carried out in non-reducing conditions, the ~57 kDa and ~42 kDa bands were not detected, whilst the ~100 kDa band became intensified (Figure 3B; line 4) suggesting that, as occurs with its homologue [18], these two polypeptides were likely to remain bound by disulphide bridges formed between the cysteines contained in their sequences.

To evaluate *Pv*RON1 presence in DRMs, a parasite lysate was solubilized at different temperatures and used for rabbit sera recognition assays. Consistent with the results that recognized the protein in the parasite lysate (Figure 3B; Line 2), polyclonal antibodies detected bands at ~100 kDa, ~57 kDa and ~42 kDa in the pellet soluble fraction at 4°C, and in the treatment soluble fraction at 37°C (Figure 3B; lines 8 and 10); these bands disappeared when the pellet was treated with the non-ionic detergent at 37°C (Figure 3B; line 12). Interestingly, sera revealed bands in the soluble fraction at 4°C (Figure 3B; line 6), which can be attributed to the high protein expression levels during schizont stage. Together, these results led to confirming that *PvR*ON1 antigen was associated with DRMs, differing from what has been reported in the DRM proteome study [15].

### Localization in rhoptries

**Figure 4:**
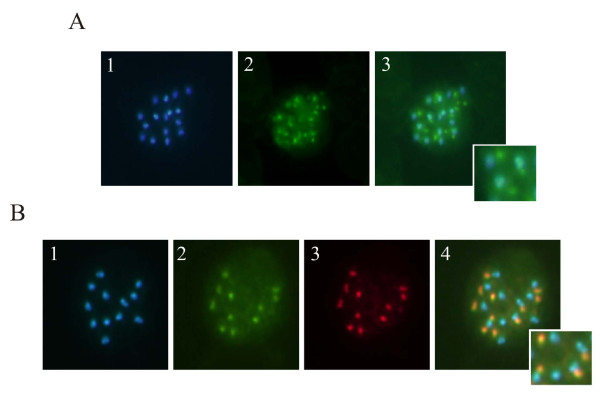
**Sub-cellular localization of the *Pv*RON1 protein in mature schizonts using fluorescence microscopy **. (A) Apical localization of *Pv*RON1 protein. (1) Nuclei stained with DAPI; (2) Detection with anti-*Pv*RON1; (3) Overlapping of the two recognition patterns. (B) Double stained assay. (1) Nuclei stained with DAPI. (2) Recognition by anti-*Pv*RON1. (3) Detection of *Pv*RhopH3. (4) Overlapping of 1, 2 and 3 images.

An immunofluorescence assay was carried out on a blood smear of *P. vivax-*infected RBCs for assessing the protein's subcellular localization in the parasite. Figure 4A shows *Pv*RON1 distribution in mature schizonts as a punctuated fluorescence pattern, which is characteristic of apical organelle proteins (rhoptries and micronemes). Alternatively, double labeling was carried out using anti-*Pv*RhopH3 mice antibodies (rhoptry bulb marker) and anti-*Pv*RON1 rabbit antibodies (Figure 4B). Overlapping images showed partial localization of both proteins, supporting the fact that *Pv*RON1 antigen could be forming part of the rhoptry neck, as occurs with *P. falciparum *ASP protein [56].

### *Pv*RON1 antigenicity

**Figure 5:**
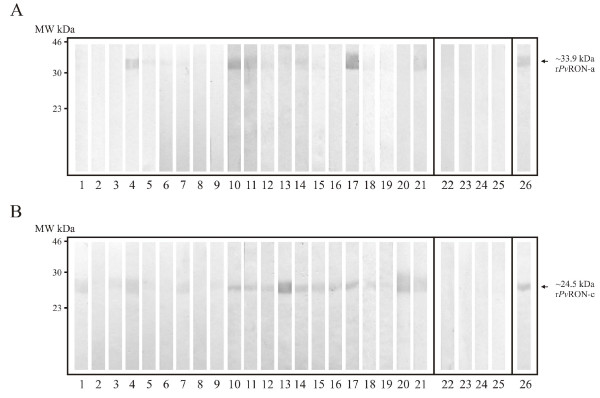
**Western blot analysis showing the recognition of *Pv*RON1 recombinant fragments by human sera **. (A-B) r*Pv*RON1-a and r*Pv*RON1-c detection. Lines 1-21, detection with sera from *P. vivax*-infected patients; Lines 22-25, recognition by control patients' sera; Line 26, detection of the protein using anti-polyhistidine monoclonal antibody.

Two fragments located towards the N- and C-termini of the *pvron1 *gene were cloned, transformed and expressed as recombinants proteins. Each purified protein's antigenicity was evaluated by Western blot using sera from 20 patients living in *P. vivax *malarial endemic regions in Colombia and who had had active episodes of the disease. Sera from four individuals who had never suffered from malaria were used as negative controls. All individuals signed an informed consent after receiving detailed information regarding the study goals. Most sera, except for that from controls, presented reactivity towards r*Pv*RON1-a and r*Pv*RON1-c (Figure 5A and 5B) fragments, suggesting that *Pv*RON1 is expressed during natural malarial infection and that it can also trigger antibody responses in the host. It has been demonstrated that antibodies directed against antigenic proteins can inhibit parasite-host interaction [21] and, therefore, these have been considered as potential candidates in designing a vaccine against malaria. All the above supports the notion that *Pv*RON1 may be a promising candidate for further trials aimed at assessing its immunogenicity and protection-inducing ability in the *Aotus *monkey model.

## Conclusions

This study has described the identification and characterization of a DRM-associated rhoptry protein. Taking into account the multiple particularities of *Pv*RON1 identified here (i.e. the gene's transcription, the protein's expression towards the end of the intraerythrocyte lifecycle and its localization and the broad recognition presented by sera from individuals infected with *P. vivax*), it can be suggested that this protein is a good vaccine candidate. The presence of secretory and GPI-anchor signals and a domain probably involved in invasion also suggested that the *Pv*RON1 antigen could play a functional role during RBC invasion; further immunogenicity and protection-inducing ability studies are thus needed in the *Aotus *experimental model to confirm the value of this protein or its products, as components of a multi-epitope, subunit-based vaccine against *P. vivax *malaria.

## Competing interests

The authors declare that they have no competing interests.

## Authors' contributions

DAMP designed experiments, analyzed data and wrote the initial manuscript. MM carried out molecular biology and immunochemical assays. MEP and MAP evaluated and coordinated the assays, and corrected the final manuscript. All authors read and approved the final manuscript.
